# LanceOtron: a deep learning peak caller for genome sequencing experiments

**DOI:** 10.1093/bioinformatics/btac525

**Published:** 2022-07-22

**Authors:** Lance D Hentges, Martin J Sergeant, Christopher B Cole, Damien J Downes, Jim R Hughes, Stephen Taylor

**Affiliations:** MRC WIMM Centre for Computational Biology, MRC Weatherall Institute of Molecular Medicine, University of Oxford, Oxford OX3 9DS, UK; MRC Molecular Haematology Unit, MRC Weatherall Institute of Molecular Medicine, University of Oxford, Oxford OX3 9DS, UK; MRC WIMM Centre for Computational Biology, MRC Weatherall Institute of Molecular Medicine, University of Oxford, Oxford OX3 9DS, UK; MRC Molecular Haematology Unit, MRC Weatherall Institute of Molecular Medicine, University of Oxford, Oxford OX3 9DS, UK; MRC WIMM Centre for Computational Biology, MRC Weatherall Institute of Molecular Medicine, University of Oxford, Oxford OX3 9DS, UK; MRC Molecular Haematology Unit, MRC Weatherall Institute of Molecular Medicine, University of Oxford, Oxford OX3 9DS, UK; MRC WIMM Centre for Computational Biology, MRC Weatherall Institute of Molecular Medicine, University of Oxford, Oxford OX3 9DS, UK; MRC Molecular Haematology Unit, MRC Weatherall Institute of Molecular Medicine, University of Oxford, Oxford OX3 9DS, UK; MRC WIMM Centre for Computational Biology, MRC Weatherall Institute of Molecular Medicine, University of Oxford, Oxford OX3 9DS, UK

## Abstract

**Motivation:**

Genome sequencing experiments have revolutionized molecular biology by allowing researchers to identify important DNA-encoded elements genome wide. Regions where these elements are found appear as peaks in the analog signal of an assay’s coverage track, and despite the ease with which humans can visually categorize these patterns, the size of many genomes necessitates algorithmic implementations. Commonly used methods focus on statistical tests to classify peaks, discounting that the background signal does not completely follow any known probability distribution and reducing the information-dense peak shapes to simply maximum height. Deep learning has been shown to be highly accurate for many pattern recognition tasks, on par or even exceeding human capabilities, providing an opportunity to reimagine and improve peak calling.

**Results:**

We present the peak calling framework LanceOtron, which combines deep learning for recognizing peak shape with multifaceted enrichment calculations for assessing significance. In benchmarking ATAC-seq, ChIP-seq and DNase-seq, LanceOtron outperforms long-standing, gold-standard peak callers through its improved selectivity and near-perfect sensitivity.

**Availability and implementation:**

A fully featured web application is freely available from LanceOtron.molbiol.ox.ac.uk, command line interface via python is pip installable from PyPI at https://pypi.org/project/lanceotron/, and source code and benchmarking tests are available at https://github.com/LHentges/LanceOtron.

**Supplementary information:**

Supplementary data are available at *Bioinformatics* online.

## 1 Introduction

Genomic elements such as enhancers, promoters and boundary elements dictate gene expression in a cell-type-specific manner (Klein and Hainer, 2020), and the high-resolution maps of these elements are composed of integrated data from genome sequencing experiments. The accurate extraction of biologically meaningful data from such assays provides the foundations of current functional genomics research and is critical to understanding gene regulation in health and disease. Genome sequencing experiments like ATAC-seq, ChIP-seq and DNase-seq are processed in a similar fashion: enriched DNA fragments are sequenced, aligned to the genome, and areas enriched for these fragments are recorded. These data appear as tracks of analog signal across genomic coordinates and increases in fragment density at true-positive biological events are called ‘peaks’ because of the characteristic pattern of fragments produced in these areas. Besides these regions, enrichment also occurs due to biases and noise in the experimental procedures (Park, 2009) or systematic mapping errors common to areas of low complexity (Amemiya *et al.*, 2019). Creating algorithms that can distinguish peaks from such experimental and computational noise and are robust across methodologies, sequencing depth, diverse tissue types and chromosomal structure has remained a challenge.

Traditionally, real peaks are distinguished from noise using statistical tests that compare enrichment from the region to the background, which is assumed to consist of a signal generated randomly. Peak callers simplify the complex analog signal of a region into a single value (maximum height) that is used with a distribution to calculate a *P*-value. While the Poisson distribution models this better than other distributions (Thomas *et al.*, 2017), the background is in fact non-random (Wilbanks and Facciotti, 2010), appearing at increased levels in areas of open chromatin (Auerbach *et al.*, 2009), at sites with inherent sequence bias and over regions of varying copy number (Vega *et al.*, 2009). This must be considered when reviewing significance from statistical peak callers, as misclassification will occur at a higher rate than the *P*-value suggests. Relying solely on these significance scores may lead to high false-positive rates, but also leaves room for potential false negatives, with the ratios of these errors depending on the parameters selected. Exacerbating these issues, default settings are routinely used which reduces accuracy nearly 10% on average compared to tuned parameters (Hocking *et al.*, 2017). With these tools, errors may be reduced by using matched negative controls (also known as input tracks) to calculate the level of background noise, though this increases the time and costs of the experiment. While peak callers such as MACS2 (Zhang *et al.*, 2008) do not strictly require negative control tracks, forgoing them may sacrifice performance (Stanton *et al.*, 2017). Input tracks do control for some experimental bias but are still sensitive to chromatin activity (Auerbach *et al.*, 2009), making statistical tests more prone to false negatives. Critically, this may cause the exclusion of peaks found in the most active areas of the genome, such as transcription start sites (TSSs) or promoters.

To address the well-known problems of peak callers, analysis pipelines employing quality control steps are common. The Encyclopedia of DNA Elements (ENCODE) consortium hosts numerous chromatin profiling assay datasets (Davis *et al.*, 2018; ENCODE Project Consortium, 2012) and has developed a robust set of guidelines including recommendations for input controls, sequencing depth, library complexity and exclusion list regions where mapping errors are more prone to occur (Landt *et al.*, 2012). Multiple replicates are encouraged, and procedures exist for combining peak calls for the most efficient reduction in error such as using Irreproducible Discovery Rate (IDR; Li *et al.*, 2011). Although these extensive measures greatly improve the reproducibility of peak calls, high-throughput visual inspection showed numerous erroneous peak calls remain (Sergeant *et al.*, 2021). This may also be linked to quality control, which is typically limited to uploading the significant regions and coverage track to a genome browser such as UCSC (Kent, 2002) or IGV (Robinson *et al.*, 2011) and manually scanning.

Though extremely time-consuming when done at scale, researchers have been shown to effectively judge the quality of peaks using a genome browser. Rye *et al.* (2011) measured peak caller performance by creating a dataset of visually verified peak calls using the UCSC genome browser, and inadvertently measured the performance of the humans in the process. They found that transcription factor motifs, known to be associated with true biological signals, were recovered more often from the manually labeled peaks than from the peak callers. Amazingly they also found that 80% of the software’s false positives could be detected even without an input control track, because the human peak callers could identify that these regions ‘lacked the expected visual appearance of a typical ChIP-seq peak’. Furthermore, while classifying regions by eye is seemingly dependent on an individual, Hocking *et al.* (2017) demonstrated a high consistency across labelers when judging peaks. Visual inspection can be a credible method for peak calling, though to do so comprehensively for an entire human genome would be nearly impossible.

Convolutional neural networks (CNNs), a class of deep learning algorithms, have been extremely successful in many general pattern detection tasks including voice recognition and image classification (LeCun *et al.*, 2015). These techniques are being applied in biology as well, especially in genomics where there is an overabundance of data available for training and analysis (Wainberg *et al.*, 2018). Tools such as DeepSea (Zhou and Troyanskaya, 2015) and Bassett (Kelley *et al.*, 2016) take genomic sequences as input and can predict regulatory genomic features with high accuracy. Proof of principle studies has also shown promise for applying these techniques to peak calling (Hocking *et al.*, 2017; Oh *et al.*, 2020).

Here, we present LanceOtron, a production-ready peak caller utilizing deep learning and deployed with a graphical user interface for integrated quality control. Complementing this, we also provide a command line interface installable from PyPI for pipeline use. LanceOtron improves upon current tools by calculating a multitude of enrichment metrics for each region being assessed and combines these with a CNN trained to recognize the characteristic shape of peaks. This model was trained with open chromatin, transcription factor and chromatin modification ChIP-seq data and achieves both high sensitivity and selectivity. Our user-friendly web tool has comprehensive filtering capabilities, built-in genome browser and automatically generated interactive charts.

## 2 Materials and methods

### 2.1 Training data

Included in the training data were punctate peaks from transcription factor ChIP-seq, broader peaks from H3K27ac and H3K4me3 histone ChIP-seq, and mixed types from ATAC-seq and DNase-seq. Training data were selected from ENCODE (Supplementary Material S1.1) then processed (Supplementary Material S1.2), resulting in 38 unique biosample types, 9 unique transcription factor ChIP-seq targets plus 2 histone ChIP-seq targets (Supplementary Table S1). These varying experiment types were included in a singular dataset to create a large collection of samples covering a breadth of peak shapes for the model to learn from (Supplementary Material S1.3). Candidate peaks were generated from these tracks (Supplementary Material S1.4) and labeled by visual inspection (Supplementary Material S1.5). Ultimately 16 990 regions were used for training: 8503 noise regions plus 8463 peaks.

### 2.2 Deep learning framework

**Fig. 1.**
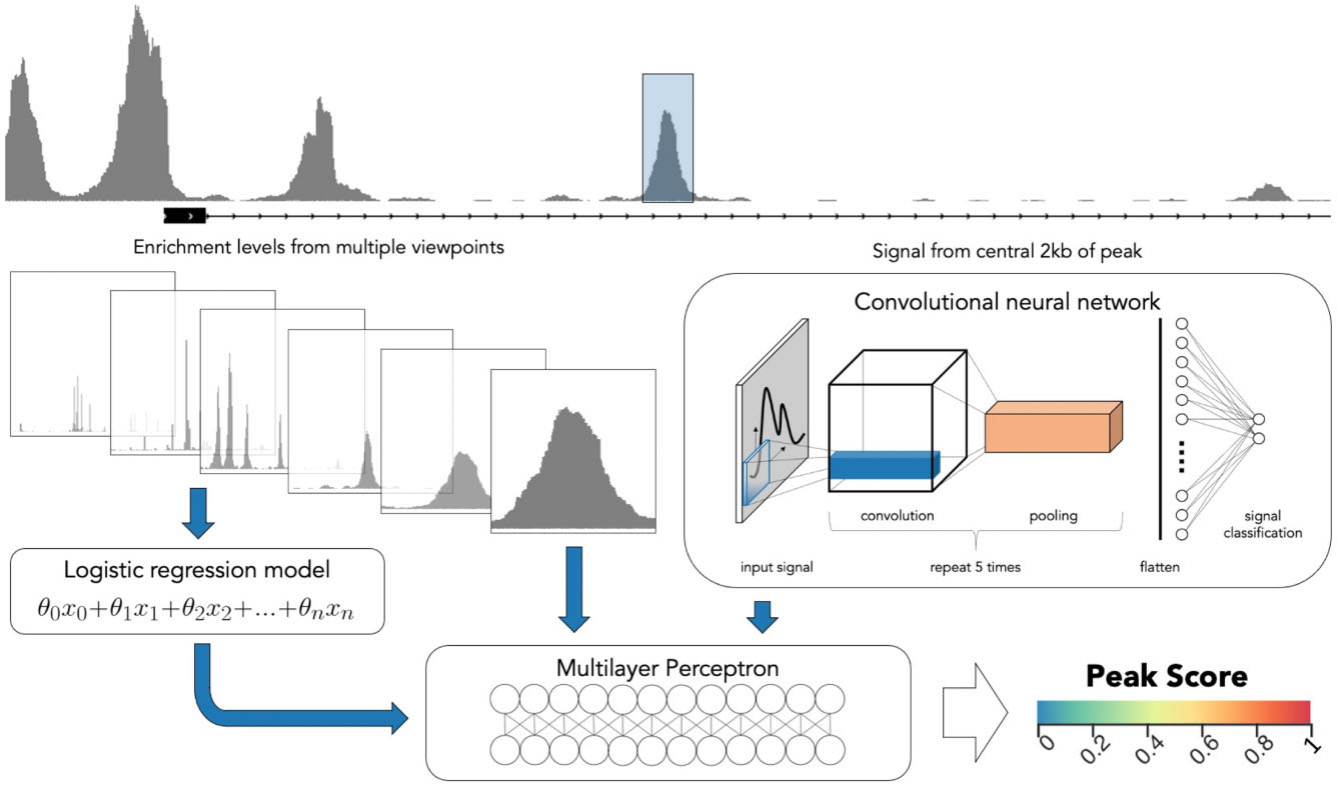
LanceOtron model overview. An indicated region has local enrichments calculated against background from 10 to 100 kb areas in 10 kb increments, plus whole chromosome (left). The enrichment values are used as inputs for a logistic regression model. Signal from the central 2 kb is fed into a CNN (right). The output from the CNN, logistic regression model and local enrichment values are all input into a multilayer perceptron (bottom), which produces the overall peak score for a given region

LanceOtron’s peak scoring algorithm is a wide and deep neural network (Cheng *et al.*, 2016) combining a CNN with local enrichment measurements. For an indicated region, a base pair resolution view of 2 kb of signal is encoded and input into LanceOtron’s CNN. The CNN then uses the relationship between the number of overlapping reads and their relative positions at all 2000 points, returning a shape score. Enrichment measurements are also taken from the maximum number of overlapping reads in a peak compared to its surroundings—chromosome-wide as well as 10–100 kb regions in 10-kb increments. The measurements are then used in a logistic regression model, which produces an enrichment score. Finally, a multilayer perceptron combines the outputs from the CNN and logistic regression models, as well as the 11 local enrichment measurements directly, to produce an overall peak quality metric called Peak Score. The comprehensive Peak Score metric is the probability of the assessed region’s signal arising from a biological event (Fig. 1).

The CNN’s design structure and hyperparameter selection were optimized using a brute force method, whereby over 5000 models were trained and tested. In addition, a specialized process for CNN training was used to maintain the independent performance of both the CNN and logistic regression models (Supplementary Material S2).

### 2.3 Lanceotron modules

Depending on the analysis to be carried out, LanceOtron’s model is employed using one of three main modules, each taking a coverage file as input and returning enriched regions with associated scores as output.

1. Find and Score Peaks, which first labels enriched regions as candidate peaks, then scores them using LanceOtron’s deep learning model.
2. Find and Score Peaks with Inputs performs the same function as the first module but additionally calculates the *P*-values of regions based on enrichment compared to a separate input control track (Supplementary Material S3).
3. Score Peaks, which does not find candidate peaks, but rather the neural network scores genomic locations provided as an additional file.

### 2.4 Candidate peak selection

To optimize resources, only enriched regions of the genome are assessed by the deep learning model. Because of the difficulty in determining a singular definition of enrichment, our candidate peak calling algorithm allows for various ways for a region to be considered enriched, with the aim of generating an overcomplete set of all possible areas of interest to present to the neural network for assessment. Signal is smoothed by calculating the rolling mean over five different window sizes, 100, 200, 400, 800 and 1600 bp. Next any coordinate where the signal is greater than fold*mean-chromosome-signal (across five different fold enrichments: 2, 4, 8, 16 and 32) is marked as enriched. Each of the 25 permutations of window size and fold threshold is considered a different definition of enrichment. The number of enrichments is tracked at each coordinate, forming a genome-wide map and regions with five or more concurring definitions of enrichment are further evaluated. If the region’s size is between 50 bp and 2 kb, it is considered a candidate peak. Regions smaller than 50 bp are discarded, and regions above 2 kb are recursively increased by an additional required enrichment definition until the region size is between 50 bp and 2 kb, or the region is considered enriched under all 25 definitions.

### 2.5 Graphical interface

A graphical web-based interface was developed to complement the candidate peak calling algorithm and improve quality control. The LanceOtron candidate peak calling algorithm was designed to identify all potentially enriched regions, score these using machine learning and return the complete dataset in a manner that can be examined and queried in its entirety—interface design and user experience decisions were made with this in mind, focusing on comprehensive filtering options and data exploration capabilities.

LanceOtron extracts genomic data from a bigwig track, which allows for peak calling and visualization to be accomplished in a single step. The LanceOtron web tool is built on the powerful MLV genome visualization software (Sergeant *et al.*, 2021), which offers important quality control features. LanceOtron’s output is automatically displayed in a genome browser with an interactive BED file, allowing for quick navigation to the areas identified by the candidate peak calling algorithm. Thumbnail images of the regions can be created and displayed in a dedicated panel, allowing hundreds or even thousands of regions to be quickly and easily scanned. In addition, the clustering/dimensionality reduction unsupervised machine learning techniques PCA (Jolliffe and Cadima, 2016), t-SNE (van der Maaten and Hinton, 2008) and UMAP (McInnes *et al.*, 2018) are included. Here, the entirety of the peak call is mapped to an interactive chart, organized across two dimensions by peak shape, where users can quickly highlight and scan subsets of their data. This allows for rapid assessment of data quality, structure and the appropriateness of the output of the algorithm for the current dataset. Users have full access to metadata and can even create their own interactive charts and displays based on any of the columns of information, giving the ability to sort and filter results *en masse* (Supplementary Videos S1 and S2).

**Fig. 2.**
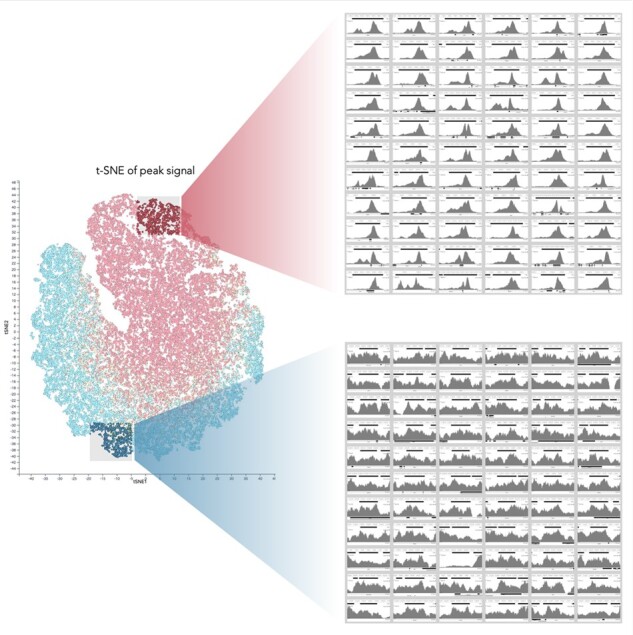
Assessing peak calls made from other tools with LanceOtron. Peak calls retrieved from ENCODE are visualized using an interactive t-SNE plot in LanceOtron’s web tool (LanceOtron 22Rv1 H3K27ac project). Screen captures from the image panel display thumbnails of high (top right) and low (bottom right) quality peaks as measured by LanceOtron’s Peak Score metric. In this experiment, roughly 33% (15 162 of 46 030) of the ENCODE-identified peaks had a <10% probability of arising from a biological event according to LanceOtron’s model

The same quality control features offered by the LanceOtron web tool can be extended for use with other tools, publications or databases using the Score Peaks module. This uploads data from an outside source and uses LanceOtron’s neural network to assess peak quality, concatenating the LanceOtron results to the original data. Using this reanalysis capability, we have found that publicly available peak calls, even following the strictest guidelines, may contain large numbers of low-quality peaks. For example, LanceOtron was used to reanalyze peaks calls from ENCODE ChIP-seq for H3K27ac from 22Rv1 prostate cancer epithelial cells (ENCSR391NPE). As part of the ENCODE IDR pipeline, two biological replicates were independently peak called and only peaks present in both were included. Using LanceOtron’s deep learning-based scoring, clustering and visualization tools, it is clear that many very low-quality peaks remain in the datasets despite requiring independent calls. Roughly, 33% (15 162 of 46 030) of the ENCODE-identified peaks had a ∼10% probability of arising from a biological event using LanceOtron’s model; large numbers of similarly low-quality peaks can be identified in many other public data sets based on statistical peak calling approaches (LanceOtron 22Rv1 H3K27ac project)(Fig. 2).

### 2.6 Peak caller benchmarking

#### 2.6.1 Simulated data

Simulated data were created using the software ChIPs (Zheng *et al.*, 2021). Parameters were estimated from ENCODE CTCF data (ENCSR692ILH) to produce paired-end FASTQ files, which were aligned with Bowtie2 (Langmead and Salzberg, 2012) on default settings. The aligned tracks were converted to bigwig format and peak called using LanceOtron. To determine the effect of peak size, potential enriched regions were placed every 10 kb across human genome hg38, increasing in size from 100 bp to 20 kb, with a step size of 100 bp and a read depth of 50 million (M) reads. To explore the effects of replicates and sequencing depth, three replicates (using different seed numbers) were generated at five different read depths: 5M, 10M, 20M, 50M and 100M. Potential sites of enrichment were found by peak calling the original CTCF dataset with LanceOtron. Intersections between datasets and Jaccard similarity coefficients were calculated using BEDTools (Quinlan and Hall, 2010).

#### 2.6.2 Labeled datasets

Benchmarking datasets were created to compare LanceOtron to MACS2, using each peak caller with default settings, with and without input tracks when available. Datasets were obtained from ENCODE (Supplementary Table S2) and explicitly not used in LanceOtron’s training data. Chromosomes were shuffled (mitochondrial and alternative mapping chromosomes were excluded), and 1 Mb was labeled for peaks or noise; regions that were not clearly either were excluded. For CTCF (ENCSR692ILH), H3K27ac (ENCSR131DVD) and H3K4me3 (ENCSR579SNM) ChIP-seq datasets, 10 chromosomes each were labeled in this manner for a total of 122 labels (55 positive peaks and 67 noise regions), 101 labels (45 positive peaks and 56 noise regions) and 224 labels (129 positive peaks and 95 noise regions), respectively. For ATAC-seq (ENCSR422SUG) and DNase-seq (ENCSR000ELW), 3 chromosomes each were labeled, resulting in 196 ATAC labels (101 positive peaks and 95 noise regions) and 224 DNase labels (114 positive peaks and 110 noise regions).

We also tested published datasets from Oh *et al.* (2020), who annotated peaks and noise for H3K27ac ChIP-seq in GM12878 cells and H3K4me3 in K562 cells for their prototype CNN-based peak caller. While their tool for labeling data and in-lab model making can still be found on GitHub, their published model is currently unavailable. We therefore compared performance against CNN-peaks by using the same datasets along with their labeled regions.

#### 2.6.3 Biological indicators

Though great efforts were employed to label megabases of the genome in numerous tracks, this still represents a small fraction of a complete human genome. To ensure that the performance measured from the labeled datasets extended genome wide, we assessed various biological indicators associated with the experiments being analyzed.

Transcription factor motif analysis was carried out by taking the top 5000 peaks from both LanceOtron and MACS2 (using input tracks), finding those regions which intersect, then using MEME (Bailey *et al.*, 2015) to discover motifs *de novo*. The MEME module FIMO (Grant *et al.*, 2011) was then used to match motifs from the peak calls produced from the different callers.

Additionally, because the transcription factor CTCF is often associated with promoters and enhancers (Holwerda and de Laat, 2013), we used GenoSTAN annotations (Zacher *et al.*, 2017) for these elements to intersect with the peaks called. Visualizing the average coverage for regions unique to the different peak callers was carried out using deepTools (Ramírez *et al.*, 2014), finding the centers of the regions and mapping the average coverage plus or minus 1 kb for the peak call.

TSSs were also used as markers associated with H3K27ac and H3K4me3 binding, as well as open chromatin—these annotations were obtained from RefTSS (Abugessaisa *et al.*, 2019). We analyzed the top 5000 peaks called from each peak caller, normalizing the regions’ size to 1 kb. This has the added benefit of being resilient to peak caller parameter changes, as the top peaks identified are unlikely to change based on parameters.

For open chromatin, we used additional published annotations from Tarbell and Liu (Tarbell and Liu, 2019), whereby they defined active areas of the genome. These were again intersected with the peak calls.

## 3 Results

### 3.1 Benchmarking LanceOtron

#### 3.1.1 Simulated data

Simulated data showed LanceOtron was able to accurately identify peaks in broadly enriched regions (Supplementary Fig. S1A), though it tended to break them apart in multiple peaks—especially for peaks larger than 1 kb (Supplementary Fig. S1B). Protein complexes actively tracking along chromatin, such as the histone marks H3K36me3, H3K79me2, represent very different biological processes with distinct distributions of signals. For calling the start and end positions of large enrichment blocks produced by these processes, we recommend using a broad peak caller.

When comparing multiple replicates across varying sequencing depths, a strong correlation was detected between replicates in a read-depth dependent manner (Supplementary Fig. S1C). Additionally, performance at sequencing depths of 10 million reads was greatly improved over 5 million reads, with some additional improvement at 20 million reads and plateauing at greater depths (Supplementary Fig. S1D).

#### 3.1.2 Labeled experimental data

We compared peak calls from transcription factor ChIP-seq, histone ChIP-seq and the open chromatin assays ATAC-seq and DNase-seq. Average peak size varied considerably from dataset to dataset (Supplementary Fig. S2A), with MACS2 having the smallest mean peak size, MACS2 with input having the largest, and LanceOtron with input and LanceOtron having the second and third largest size, respectively (Supplementary Fig. S2B). While LanceOtron with input identified important biological features at a high rate, it did so covering the smallest number of base pairs in its peak calls (Supplementary Fig. S2C). Increasing stringency beyond MACS2’s default settings did improve F1 overall performance, but at the cost of sensitivity, and was still outperformed by LanceOtron on every dataset tested; using MACS2’s broad option reduced performance over default, but using MACS2 in combination with LanceOtron (via the Score Peaks module) improved performance (Supplementary Fig. S3). The best overall performance was achieved using LanceOtron with Input—for a numerical listing of performance benchmarks for the labeled datasets see Supplementary Table S3.

#### 3.1.3 Transcription factor ChIP-seq

**Fig. 3.**
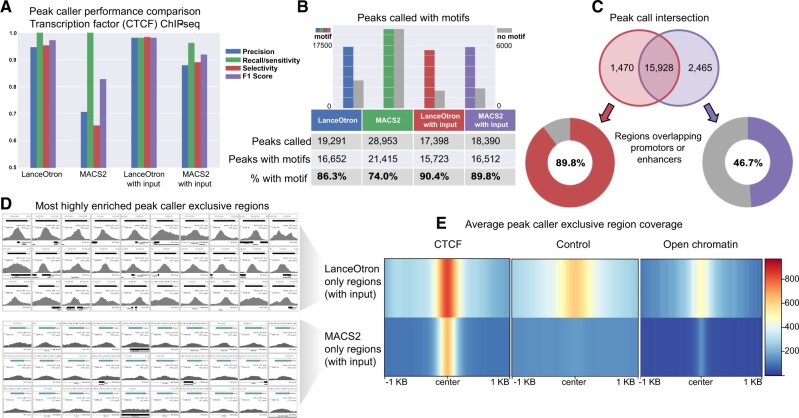
Benchmarking LanceOtron against MACS2 for peak calling transcription factor ChIP-seq. (**A**) Model performance metrics using labeled genomic regions of an ENCODE CTCF ChIP-seq dataset. (**B**) Comparing the number of motifs contained in peak calls generated from LanceOtron and MACS2. (**C**) Venn diagram of peak calls from LanceOtron and MACS2; regions that did not intersect were assessed for overlap with promotors or enhancers. (**D**) Thumbnail images from the most highly enriched regions called exclusively by either LanceOtron (top) or MACS2 (bottom). (**E**) Average coverage of the regions called exclusively by either LanceOtron (top) or MACS2 (bottom) for CTCF experimental track, control track and DNase-seq open chromatin track

Transcription factor ChIP-seq was assessed using CTCF in spleen primary cells. When no input control track was used, both LanceOtron and MACS2 achieved perfect sensitivity, detecting all labeled peaks in the dataset, but MACS2 had far lower selectivity and overall F1 score. With input, LanceOtron outperformed MACS2 in precision, recall/sensitivity, selectivity and F1 score. Comparing across peak call types, LanceOtron without input actually achieved higher scores than MACS2 with input across all metrics, though the highest F1 score for this dataset was obtained when LanceOtron was used with an input track (LanceOtron spleen CTCF projects: without input; with input) (Fig. 3A).

Motif analysis was also carried out for this dataset. LanceOtron called fewer peaks with motifs than MACS2 but called fewer peaks in total, resulting in a larger percentage of the overall peak call containing motifs: 86.3% for LanceOtron versus 74.0%% for MACS2; with input LanceOtron found 90.4% versus MACS2 with 89.8% (Fig. 3B). This trend was also seen when performing motif enrichment analysis on other transcription factors, with LanceOtron generally finding a larger percentage of peaks with motifs (Supplementary Table S4).

We further investigated the differences between LanceOtron with input and MACS2 with input peak calls, finding 1470 LanceOtron only and 2465 MACS2 only regions. We additionally found 89.8% of peaks exclusively called with LanceOtron overlapped with promoters or enhancers compared to just 46.7% of MACS2 only peak calls (Fig. 3C). When visualizing the top enriched regions called exclusively by each peak caller, LanceOtron’s peaks have strikingly more signal than MACS2 (Fig. 3D). This was also seen when inspecting the average signal of the exclusive peak calls; MACS2 only regions were found in areas with less surrounding signal, containing peaks which were narrower and with very low enrichment compared to LanceOtron only peaks. It seems that the MACS2-only regions are a sporadic sampling of the numerous peaks close to noise found throughout the genome; however the peaks that MACS2 missed are relatively strongly enriched. These missing peaks are excluded by MACS2 because of the increase in control signal, however, some increased signal from the control track is expected when the region is found in areas of open chromatin (Auerbach *et al.*, 2009), which can be seen associated with the LanceOtron only peaks (Fig. 3E). This is further exemplified by when peak calling the transcription factor SRF in GM12878 cells, an element known to bind with other cofactors at TSSs. Here, LanceOtron peaks were found to intersect TSSs more often than MACS2 peaks (35.4% of the peak call versus 20.4% when using input for both peak callers; Supplementary Table S5). Of the peaks MACS2 missed, 1211 were found within 1 kb of TSSs (LanceOtron GM12878 SRF with input), and are most prominently associated with genes involved in cell cycle, DNA replication, DNA repair and metabolism of RNA (Supplementary Fig. S4)—genes which are closely related to SRF’s function (Onuh and Qiu, 2021).

#### 3.1.4 Histone ChIP-seq

**Fig. 4.**
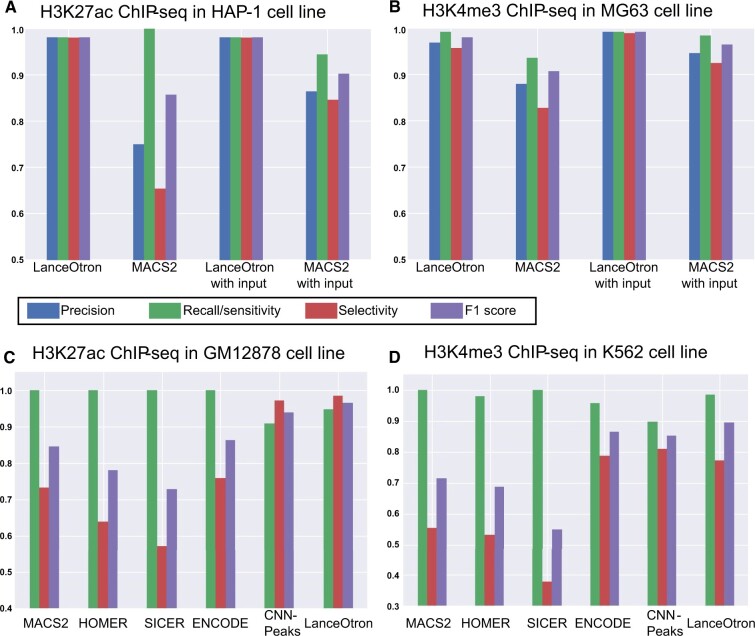
Benchmarking LanceOtron against MACS2 for peak calling histone ChIP-seq. (**A**) Model performance metrics using 10 Mb of labeled genomic regions of ENCODE ChIP-seq datasets for H3K27ac in HAP-1 cell line, and (**B**) H3K4me3 in MG63 cell line. (**C**) ChIP-seq dataset labeled by Oh *et al.* for H3K27ac in GM12878 cell line and (**D**) H3K4me3 in K562 cell line

Histone ChIP-seq was assessed using H3K27ac in HAP-1 cells and H3K4me3 in MG63 cells. For H3K27ac, the top sensitivity was achieved with three peak calls: LanceOtron, both with and without input, and MACS2 without input. LanceOtron outperformed MACS2 in the remaining metrics of precision, selectivity and F1 score. The same performance was achieved both with and without input for the LanceOtron peak calls, highlighting the power of its deep neural network (LanceOtron HAP-1 H3K27ac projects: without input; with input; Fig. 4A). In the H3K4me3 dataset, specificity was equal between LanceOtron and MACS2 with input, and LanceOtron outperformed MACS2 across all peak call types for the remaining metrics (LanceOtron MG63 H3K4me3 projects: without input; with input; Fig. 4B).

We also tested LanceOtron’s performance against published datasets for H3K27ac ChIP-seq in GM12878 cells and H3K4me3 in K562 cells. LanceOtron achieved the highest overall F1-score for both histone datasets, besting CNN-peaks as well as MACS2, HOMER (Heinz *et al.*, 2010), SICER (Zang *et al.*, 2009) and ENCODE’s IDR method. These results were consistent with our in-house labeled data, with legacy peak callers performing slightly better on sensitivity, and LanceOtron outperforming on selectivity and F1 score [LanceOtron GM12878 H3K27ac project (Fig. 4C); LanceOtron K562 H3K4me3 project (Fig. 4D)].

To further investigate the histone mark ChIP-seq peak calls, we counted the number of TSSs overlapping with the peak calls. For H3K27ac, LanceOtron performance was very similar with and without input, increasing from 2806 to 2812 peaks when the input track was included. Both LanceOtron peak calls had more overlap with TSSs than MACS2, which had 2428 and 2607 with input. We observed similar results for the H3K4me3 data, with LanceOtron finding 3472 peaks intersecting TSSs, increasing slightly to 3501 with input control. MACS2 had better performance without input, though not reaching LanceOtron levels, at 3318 and decreasing down to 2491 with input (Table 1).

**Table 1.** LanceOtron and MACS2 peak call comparison for TSSs and for active regions in open chromatin

	LanceOtron	MACS2	LanceOtron with input	MACS2 with input
% top H3K27ac ChIP-seq in HAP-1 peaks overlapping TSSs (count)	56.1% (2806/5000)	48.6% (2428/5000)	56.2% (2812/5000)	52.1% (2607/5000)
% top H3K4me3 ChIP-seq in MG63 peaks overlapping TSSs (count)	69.4% (3472/5000)	66.4% (3318/5000)	70.0% (3501/5000)	49.8% (2491/5000)
% top ATAC-seq in MCF-7 peaks overlapping TSSs (count)	44.4% (2218/5000)	21.9% (1096/5000)		
% top DNase-seq in A549 peaks overlapping TSSs (count)	43.3% (2164/5000)	22.7% (1133/5000)		
% ATAC-seq peaks in active regions (count)	12.6% (628/5000)	7.5% (377/5000)		
% DNase-seq peaks in active regions (count)	12.1% (607/5000)	9.5% (477/5000)		

Percentages and counts of peaks intersecting TSSs are given for 5000 regions of LanceOtron and MACS2 peak calls, selected for being most enriched (highest *q*-value or peak score for LanceOtron and MACS2, respectively). Percentages and counts are also shown for open chromatin peaks found in active areas of the genome.

#### 3.1.5 ATAC-seq and DNase-seq

**Fig. 5.**
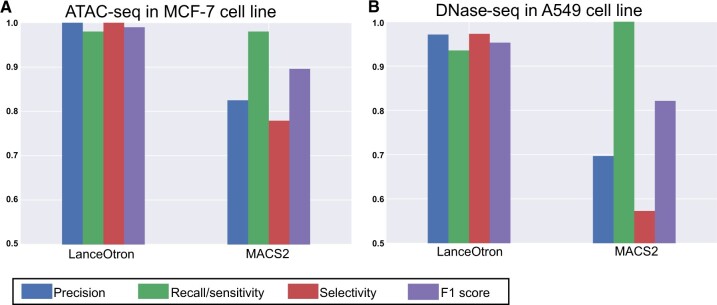
Benchmarking LanceOtron against MACS2 for open chromatin. (**A**) Model performance metrics using labeled genomic regions of an ENCODE ATAC-seq dataset in MCF-7 cell line and (**B**) DNase-seq in A549 cell line

ATAC-seq assessment was carried out using the MCF-7 cell line. LanceOtron outperformed MACS2 across all metrics (LanceOtron MCF-7 ATAC-seq project; Fig. 5A). Results were similar for our in-house DNase-seq data in the A549 cell line. MACS2 outperformed LanceOtron for recall/sensitivity but had a very high false-positive rate. Consequently, LanceOtron outperformed MACS2 on precision, sensitivity and F1 score (LanceOtron A549 DNase-seq project; Fig. 5B). As with the histone datasets, we also intersected the top 5000 peaks with TSSs. LanceOtron’s top peaks had just under double the number of intersections with TSSs compared with MACS2 for DNase-seq (2164 versus 1133) and just over double for ATAC-seq peaks (2218 versus 1096; Table 1).

We also compared peak calling performance on GM12878 cells for ATAC-seq (ENCFF576DMC) and DNase-seq (ENCSR000EMT) using outside published annotations (Tarbell and Liu, 2019). The number of peaks called for these datasets was considerably different from each peak caller. For ATAC-seq, 60 962 peaks were called using LanceOtron and 127 304 peaks for MACS2; DNase-seq, 25 183 peaks were called using LanceOtron and 85 607 peaks for MACS2. For both ATAC-seq and DNase-seq, a higher percentage of the LanceOtron peak call intersected the annotations than MACS2 (ATAC-seq: 12.6% versus 7.5%; DNase-seq: 12.1% versus 9.5%; LanceOtron GM12878 projects: ATAC-seq; DNase-seq; Table 1).

## 4 Discussion

LanceOtron is a deep learning-based peak caller for genomic signal analysis, with a full user-friendly interface designed for interrogation of large datasets. In our performance comparison, it outperformed the current gold standard algorithm, MACS2, in each of our experiments, and when expanded out to published benchmarks it also surpassed CNN-Peaks, HOMER, SICER and even ENCODE’s IDR process for measuring consistency across replicated peaks in multiple samples. LanceOtron’s CNN, trained on open chromatin, transcription factor and chromatin modification data, learns the shape of the signal and uses this in combination with enrichment calculations to identify biologically relevant regions. Traditional peak callers return only those regions which cross a high statistical threshold. When using LanceOtron’s candidate peak calling algorithm however, all enriched regions above a relatively low threshold are returned, along with their associated Peak Scores, *P*-values, heights, widths and other properties. This makes LanceOtron akin to an automated annotation tool, returning a greater breadth of data about the experiment. Peak calls can be quickly and accurately made using LanceOtron’s Peak Score. This summary metric predicts peak or noise classifications at high probabilities, making it largely invariant to parameter changes (88% of candidate peaks were assigned Peak Scores of less than 0.1 or greater than 0.9; Supplementary Fig. S5). Ultra-refined peak calls can also easily be made using LanceOtron’s comprehensive visualization, filtering and data handling to generate output datasets with defined characteristics.

LanceOtron peaks were shown to be enriched for the CTCF binding motif at a higher percentage than MACS2. Furthermore, peaks uniquely identified by LanceOtron were enriched for enhancers or promoters, providing additional biological evidence that the differences in peaks called by LanceOtron are actually improvements over traditional analysis. Experiments such as these producing punctate peaks are perhaps the best-suited datasets for statistical peak callers. This means they should make fewer errors, especially concerning false negatives, however, in our testing some real peaks appeared to be absent from the MACS2 dataset. Inspection of the DNase-seq track made it clear that many of the regions missed by MACS2 were in regions of open chromatin. These areas sonicate more readily (Auerbach *et al.*, 2009) and are known to have increased signal in input tracks, however, this increase in control signal did not preclude these regions from being recognized using LanceOtron as it did for MACS2. The loss of these regions in the MACS2 analysis is likely due to a combination of its reliance on the signal in the input tracks to judge peak quality, and its high *P*-value threshold used to better reduce false positives genome wide, which it does at the cost of sensitivity in active regions of the genome. As an example, when analyzing ChIP-seq for the SRF protein over gene promoters, MACS2 showed a substantial loss of genes (1211 peaks within 1 kb of TSSs) known to be linked with SRF’s function.

MACS2 performance was strongly affected by input track availability: for the experiments tested with the input track present and absent, the mean percentage change of the performance metrics between track types was 7.5% for MACS2. This is in contrast to LanceOtron, which maintained performance without an input track, with a mean percentage change of 1.3%; LanceOtron without input even achieved higher overall F1 scores than MACS2 with input for every dataset where this comparison was available (Supplementary Table S3). The inclusion of input tracks may behave as a double-edged sword for MACS2, as this improved performance may limit the ability to detect peaks in the most active areas of the genome (such as TSSs), as it did with SRF ChIP-seq.

Perhaps the natural choice of tool to benchmark LanceOtron against was CNN-Peaks, however, the majority of our performance reviews focused on MACS2. This was because the published model from CNN-Peaks was no longer available, and MACS2 represented the next highest performing tool. ENCODE’s processing pipeline was another top performer, however, this method also uses the MACS2 algorithm. ENCODE employs post-processing after peak calling (which in theory could be used with any tool, not exclusively MACS2), but even without these additional steps, LanceOtron achieved a higher F1-score. In many ways, MACS2’s enduring popularity is linked to its ease of use: it works as expected according to its documentation, installs easily across a range of systems and continues to be well supported by its developers. This gives it an advantage over proof-of-principle methods like CNN-Peaks or even well-established data processing pipelines such as ENCODE’s. Inspired by this, we made LanceOtron available as both a web tool and a command line tool available from PyPI, installable by typing a single line of code in the terminal (pip install lanceotron) from most any computer running a recent version of Python.

The average time to perform a peak call on the 13 datasets benchmarked here was just over an hour (mean time 67 min, standard deviation 11 min) *within a web browser—*this includes the time taken to generate the interactive charts and upload the coverage track into the genome browser. The speed that LanceOtron can carry out analysis, requiring only a basic bigwig track and using a web interface, has obvious benefits for routine use and is even applicable as part of a manuscript review process. While a session of data is often provided during review, this is seldom utilized due to time constraints, bioinformatic complexity and potentially the need of high-performance computing facilities. LanceOtron remedies this, providing a convenient outlet for group leaders, bench biologists and bioinformaticians alike to visualize and assess datasets from internal or external sources. In addition, peak calls made with LanceOtron can easily be made public for assessment by reviewers and colleagues directly, as they have been here. LanceOtron can even be applied to datasets that have already been analyzed by other methods; re-calling MACS2 datasets with LanceOtron’s Score Peaks model improved performance on every dataset benchmarked here (Supplementary Fig. S3 and Table S3).

A strength of using supervised machine learning approaches is that analysis can improve as more training data is added to the model; as our user base grows, we can refine our peak calls even further. Our focus to date has been on the most commonly used experiments where we believed there was the greatest potential for improvement. However, unlike hardwired statistical algorithms, CNN-based algorithms can easily be trained to deal with new signal types and distributions not covered in the original training sets. The same architecture can potentially be used to learn different types of genomics data, for example CAGE TSS signals or methylomics which are currently challenging to extract signal from noise; exemplifying this, LanceOtron has even been adapted for analyzing base pair resolution chromosome conformation capture (Hua *et al.*, 2021).

In summary, LanceOtron is a powerful peak caller and analysis tool for use across a wide range of epigenetic marks. Testing with numerous datasets and data types, LanceOtron outperformed the industry standard MACS2 as well as other tools published as best in class. It is designed to accommodate current workflows as a visualization, annotation, filtering and peak calling tool, leveraging a powerful deep learning neural network to use peak shape information alongside enrichment data.

## Supplementary Material

btac525_Supplementary_Data

## Data Availability

The data underlying this article are available in the article and in its online supplementary material.
