# Supplementary material for: LanceOtron: a deep learning peak caller for genome sequencing experiments
Source: Bioinformatics. 2022 Jul 22;38(18):4255–63. doi: 10.1093/bioinformatics/btac525 (PMC9477537; doi:10.1093/bioinformatics/btac525)
Supplement: btac525_Supplementary_Data [file btac525_supplementary_data.pdf]

Supplementary material for *LanceOtron: a deep learning peak caller for genome sequencing experiments*

S1 Training data acquisition and processing

S1.1 Selecting ENCODE experiments

To generate a complete list of experiments which met our specifications we used ENCODE's REST API (scripts and outputs available on GitHub). We filtered the results to samples which were "released" status at the time of search inquiry and aligned to human reference genome hg38 as BAM files; for H3K27ac, H3K4me3, and transcription factor ChIP-seq experiments, the availability of a corresponding control track was also required. While infrequent, samples were excluded if ENCODE metadata did not include information on single-end versus paired-end sequencing. The number of samples meeting these criteria was 3,902 (74 ATAC, 911 DNase, 305 H2K27ac, 463 H3K4me3, 2,149 transcription factor samples).

From this 10 paired-end datasets were sampled at random from each experiment type, except in H3K4me3 experiments where only 6 samples available were paired-end, and so 4 single end experiments were included.

S1.2 Data processing

Each BAM file was downloaded directly from ENCODE, along with the corresponding control BAMs for H3K27ac, H3K4me3, and transcription factor ChIP-seq experiments. If multiple replicates of the control experiments existed, only the first listed in ENCODE's database was used for analysis. BAM files were sorted and indexed using Samtools(Li *et al.*, 2009) 1.3 (`samtools sort filename.bam` and `samtools index filename.bam.sorted` commands respectively). Bigwig file coverage maps were created from the BAM files using deepTools(Ramírez *et al.*, 2014) version 3.0.1 commands: `bamCoverage --bam filename.bam.sorted -o filename.bw --extendReads -bs 1 --normalizeUsing RPKM` for paired-end sequenced experiments. For single-end sequenced experiments the average fragment length was obtained from ENCODE and used with the --extendReads flag, making the command: `bamCoverage --bam filename.bam.sorted -o filename.bw --extendReads averageFragmentLength -bs 1 --normalizeUsing RPKM`.

S1.3 Combining datasets

The choice of dataset structure is closely linked to the model used to interpret the data. It was decided for our purposes that combining datasets from ATAC-seq, ChIP-seq, and DNase-seq would better benefit our model design. Deep learning requires very large datasets, which are used to establish the properties of the inputs. By giving the model ATAC-seq, ChIP-seq, and DNase-seq the model has a greater breadth and quantity of

examples to understand the input space. This is in contrast to creating individual models for each experiment type, which would still learn roughly the same features, but would have fewer samples to train from. In addition, experiments may have a mix of peak shapes, with some types appearing more frequently than others, and combining datasets also allows for reduction in misclassifying rare peak types.

S1.4 Randomly selecting regions to label

Putative peak calls were carried out on all datasets, followed by classification as either peak or noise based on visual inspection. Coordinates for the regions being assessed were determined three ways. The MACS2 peak caller was used on default settings, `macs2 callpeak -t filename.bam.sorted -c control_filename.bam.sorted -n sample_label -f BAM -g hs -B -q 0.01` for H3K27ac, H3K4me3, and transcription factor ChIP-seq datasets. For ATAC-seq and DNase-seq, which lack control tracks, the following command was used: `macs2 callpeak -t filename.bam.sorted -n sample_label -f BAM -g hs -B -q 0.01`. The second and third peak call methods focused on labeling regions based on their fold enrichment compared to the mean signal. Coverage maps of sequenced reads were first smoothed by applying a rolling average of a given window size. If this smoothed signal was greater than the mean multiplied by a fold enrichment threshold, the coordinate was marked as enriched; adjacent enriched regions were then merged. Methods two and three used five smoothing windows at different base pair (bp) resolutions (100 bp, 200 bp, 400 bp, 800 bp, 1600 bp) as well as five different enrichment thresholds (1, 2, 4, 8, 16). Method two compared the smoothed signal to the mean of chromosome-wide signal multiplied by fold enrichment. Method three was similar except the smoothed signal was compared to either the mean of the chromosome, surrounding 5 kb, or surrounding 10 kb, whichever value was highest (i.e. `max[chromosome mean, 5 kb mean, 10 kb mean]`) multiplied by fold enrichment.

From each dataset a 1 Mb continuous region was selected at random for each chromosome for autosomes and sex chromosomes only. If the start of the randomly selected region was near the end of the chromosome, the area considered was from that point to the chromosome end, then from the chromosome start extending out until a full 1 Mb was covered. Peaks called from all 3 methods which started within the random region were made available for labeling. For both of the mean-based methods, a peak call was made for each permutation of the smoothing window and enrichment threshold parameters, and all 25 calls were combined - this meant the presence of multiple overlapping candidate peaks in some cases. A python implementation of BEDTools(Quinlan and Hall, 2010) (pybedtools) was used to find overlapping peaks, and only one selected at random was considered for visual inspection.

S1.5 Data labeling

Only candidate regions which were obviously peaks or noise were labeled as such. Visual inspection was carried out using MLV (Sergeant *et al.*, 2021), with control tracks overlaid when available. Regions were inspected one at a time, until either 100 verified peaks were found for the dataset or all of the regions were assessed. Entire 1 Mb regions were assessed (no early stopping), with the order of chromosomes randomized. A total of

736,753 regions were labeled this way (5,016 peaks and 731,737 noise regions) covering 499 Mb. A subsample of these labeled data, alongside algorithmically labeled data (described below), were used for training the first phase of the model. Afterwards the training data was scored with the preliminarily trained model to identify any mislabeled data or misclassifications; from this process 24 peaks and 1,187 noise regions were added to the dataset. Ultimately 16,990 regions were used for training: 8,503 noise regions plus 8,463 peaks.

Additional labels were generated using an algorithm. First the raw signal was smoothed by calculating the rolling mean for the surrounding 400 bp, and any coordinate where the signal was 4-fold*mean-chromosome-signal was marked as enriched. Adjacent enriched regions were combined, and if the size was between 50 bp and 2 kb it was considered a candidate peak. Regions smaller than 50 bp were discarded, and regions above 2 kb were recursively re-evaluated at a 1-fold higher threshold until the region size was between 50 bp and 2 kb, or the region was greater than 20-fold enriched. If these candidate peaks intersected with the previously labeled peaks, these regions were then also labeled peaks, resulting in an additional 3,447 labels for a total of 8,463 peaks (ATAC-seq: 1,926; DNase-seq: 2,097; H3K27ac ChIP-seq: 1,651; H3K4me3 ChIP-seq: 1,806; transcription factor ChIP-seq: 983). Noise regions were down sampled with prioritization given to regions with the highest signal. All noise regions with a max height in the 25th percentile or greater were included (3,658), and equal numbers below the 25th percentile were randomly sampled.

Notably, the candidate peak calling algorithm LanceOtron uses closely resembles the coordinate selection process described above. This ensures that when working with real data, it is presented to the neural network in a similar fashion to the training data here.

S2 Deep learning model design and training

LanceOtron's machine learning architecture is a type of wide and deep neural network(Cheng *et al.*, 2016), combining enrichment values, logistic regression, and a CNN. The logistic regression model takes as inputs the enrichment values, while the CNN uses the 2 kb of signal centered on the region of interest. The outputs of these two models, along with the 11 enrichment values, are input into a multilayer perceptron, which outputs a peak quality metric (called Peak Score) with values ranging from 0 to 1.

The 11 enrichment values consisted of Poisson-based p-values, using maximum height and average signal, calculated from 10kb to 100kb regions in 10kb increments as well as chromosome-wide enrichment. While this is an internal model parameter, and not used for significance thresholding, we opted to use the p-value because of the increased interpretability, though numerous enrichment metrics could have been used to yield similar results. These p-values are also returned to the user as an additional calculated measurement; indeed the results of a traditional statistical peak caller could be mimicked by simply using p-values as the sole filtering metric.

Because the labeling procedure included only those regions which were obviously peaks or noise, the target values of the training data were 0 for noise, and 1 for peak. The logistic regression model was trained separately with the same training data, and all coefficients and model parameters saved. The wide and deep model was then trained with the logistic

regression component locked, and with loss distributed 70:30 to wide-and-deep-output:CNN-only-output. By penalizing the model on the CNN separately, it actively encouraged predictions from the 2 kb of signal, i.e., the shape of the peak, to be accurate in absence of enrichment information.

To determine the optimal structure and hyperparameters, a brute force method of building many models with different configurations was carried out. Model per- formance was assessed by measuring the number of correctly predicted clas- sifications of enriched regions from data unseen to the model. This was done in two stages using the RandomSearch function from the python package Keras Tuner. First, 5000 models were built varying the following components: the first convolutional layer's filter number (50-200) and filter size (2-10), the number of convolutional blocks (1-5), number of convolutional layers per block (1-5), convolutional filters used per hidden convolutional layer (20-100) and their size (2-10), pooling type (max, average, or none), dropout rate (0.0-0.5), first dense layer size (10-100), number of additional dense layers (0-3), additional dense layer size (10-100), and learning rate (0.0001-0.01). Convolutional blocks were composed of first convolutional layers, then batch normalization, followed by an optional pooling layer. Performance was robust across a range of configurations. This included models with less complexity, which required less time and computational resources. Given this, several architectural choices were made: fixing the number of convolutional blocks to four, using one convolutional layer per block, and using max pooling; the model used two additional dense layers. The search process was repeated, building another 500 models which explored the following range of component variables: the first convolutional layer's filter number (50-150) and filter size (2-10), convolutional filters used per hidden convolutional layer (50-200) and their size (2-10), dropout rate (0.0-0.5), first dense layer size (10-100), additional dense layers size (10-100), and learning rate (0.0001-0.01). The top 10 performing models were then subjected to 5-fold cross validation, and the architecture from the top performer was used.

S3 Calculating p-value from an input control track

A standard p-value assessment based on the Poisson distribution is performed when using LanceOtron's Find and Score Peaks with Inputs module, which can be used in conjunction with the peak quality metric output from LanceOtron's deep learning model. The mean signal expected from background, λ, is determined using either the mean signal in the input control track (λ_{input}) or the mean signal in the input control track plus 1 kb (λ_{1kb}), whichever is more stringent. P-values are then computed using the average count of overlapping reads (N_{ave}) within the given candidate region.

$$\text{p-value} = 1 - pPois\left(N_{ave}, \lambda = \max\left[\lambda_{input}, \lambda_{1kb}\right]\right),$$

where *pPois* is the Poisson cumulative distribution function:

$$\sum_{i=0}^{N_{ave}} \frac{e^{-\lambda}\lambda^{i}}{i!}$$

Supplementary figure 1 - Simulated data

F1 scores across peak size ranges from simulated data (**A**). While the lowest range had smaller regions for the simulated reads to be mapped, resulting in undetectably low read coverage and reduced F1 scores, the expansive regions allowed for sufficient coverage to detect nearly all potential enrichment sites. However, when looking at regions which are divided into multiple peaks (**B**), these are found much more frequently in regions larger than 1 kb.

Heatmap showing ratio of peaks found in both datasets across different replicates and sequencing depths (**C**). Strong correlation was detected between replicates in a read-depth dependent manner. At the lowest read depth (five million [5M] reads) the small number of peaks called were found in very high percentages across all other peak calls (**C**, top-most three rows), but made up a smaller percentage of the total peak calls from the higher sequencing depth calls (**C**, left-most three rows). This is as expected, since at low coverage only the strongest peaks will remain, be found in all datasets, and as coverage increases there is an increased ability to detect sites which are less frequently bound which will be absent at low depths.

Heatmap showing the Jaccard similarity coefficient between datasets (**D**). Datasets have much weaker correlation at 5M reads, have a large improvement when increased to 10M reads, a modest improvement when further increased to 20M reads, and plateauing effects from there.

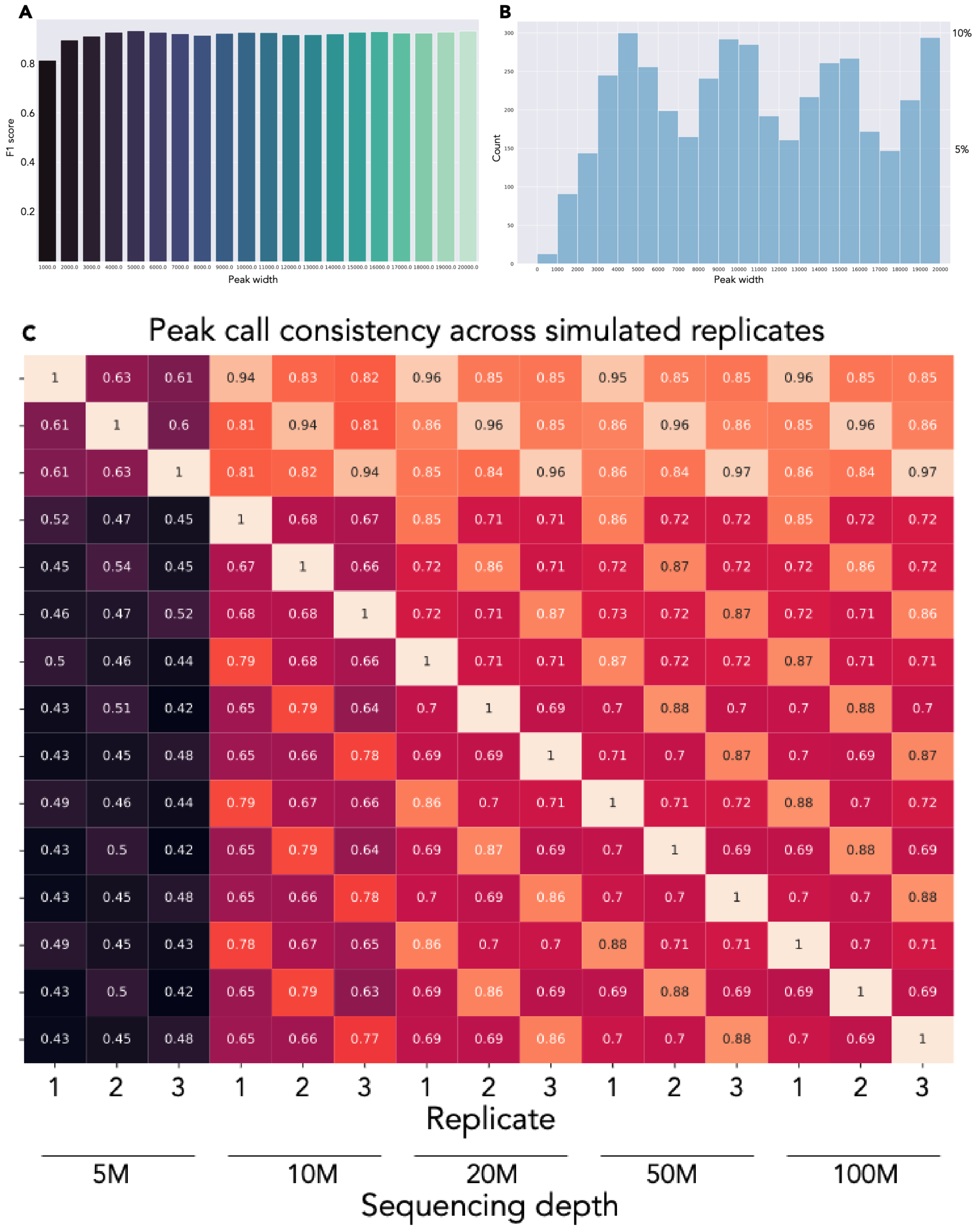
A
F1 score
0.8
0.6
0.4
0.2
Peak width
B
Count
10%
5%
Peak width
c
Peak call consistency across simulated replicates
1 0.63 0.61 0.94 0.83 0.82 0.96 0.85 0.85 0.95 0.85 0.85 0.96 0.85 0.85
0.61 1 0.6 0.81 0.94 0.81 0.86 0.96 0.85 0.86 0.96 0.86 0.85 0.96 0.86
0.61 0.63 1 0.81 0.82 0.94 0.85 0.84 0.96 0.86 0.84 0.97 0.86 0.84 0.97
0.52 0.47 0.45 1 0.68 0.67 0.85 0.71 0.71 0.86 0.72 0.72 0.85 0.72 0.72
0.45 0.54 0.45 0.67 1 0.66 0.72 0.86 0.71 0.72 0.87 0.72 0.72 0.86 0.72
0.46 0.47 0.52 0.68 0.68 1 0.72 0.71 0.87 0.73 0.72 0.87 0.72 0.71 0.86
0.5 0.46 0.44 0.79 0.68 0.66 1 0.71 0.71 0.87 0.72 0.72 0.87 0.71 0.71
0.43 0.51 0.42 0.65 0.79 0.64 0.7 1 0.69 0.7 0.88 0.7 0.7 0.88 0.7
0.43 0.45 0.48 0.65 0.66 0.78 0.69 0.69 1 0.71 0.7 0.87 0.7 0.69 0.87
0.49 0.46 0.44 0.79 0.67 0.66 0.86 0.7 0.71 1 0.71 0.72 0.88 0.7 0.72
0.43 0.5 0.42 0.65 0.79 0.64 0.69 0.87 0.69 0.7 1 0.69 0.69 0.88 0.69
0.43 0.45 0.48 0.65 0.66 0.78 0.7 0.69 0.86 0.7 0.7 1 0.7 0.7 0.88
0.49 0.45 0.43 0.78 0.67 0.65 0.86 0.7 0.7 0.88 0.71 0.71 1 0.7 0.71
0.43 0.5 0.42 0.65 0.79 0.63 0.69 0.86 0.69 0.69 0.88 0.69 0.69 1 0.69
0.43 0.45 0.48 0.65 0.66 0.77 0.69 0.69 0.86 0.7 0.7 0.88 0.7 0.69 1
1 2 3 1 2 3 1 2 3 1 2 3 1 2 3
Replicate
5M
10M
20M
50M
100M
Sequencing depth

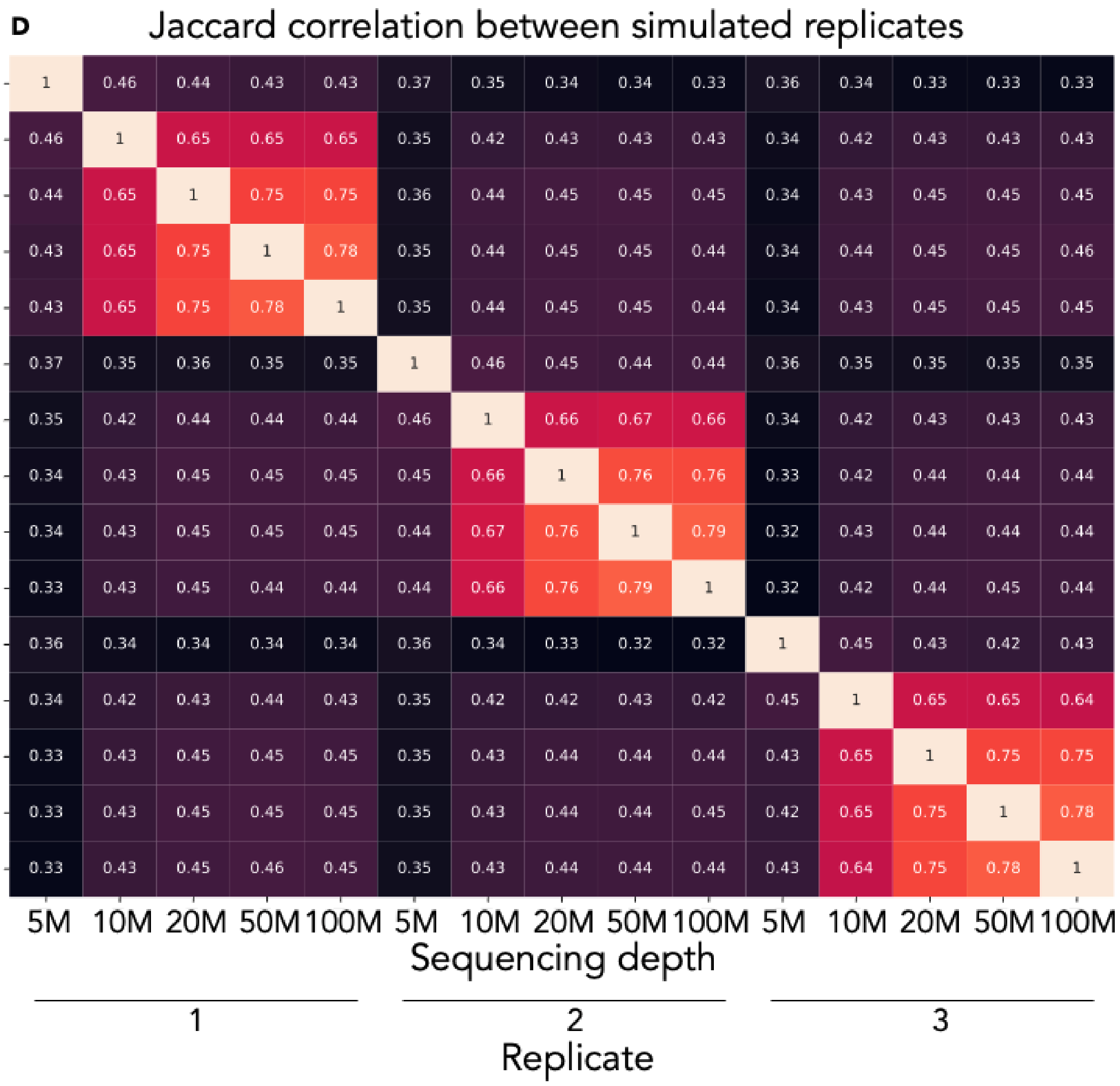
D
Jaccard correlation between simulated replicates
1 0.46 0.44 0.43 0.43 0.37 0.35 0.34 0.34 0.33 0.36 0.34 0.33 0.33 0.33
0.46 1 0.65 0.65 0.65 0.35 0.42 0.43 0.43 0.43 0.34 0.42 0.43 0.43 0.43
0.44 0.65 1 0.75 0.75 0.36 0.44 0.45 0.45 0.45 0.34 0.43 0.45 0.45 0.45
0.43 0.65 0.75 1 0.78 0.35 0.44 0.45 0.45 0.44 0.34 0.44 0.45 0.45 0.46
0.43 0.65 0.75 0.78 1 0.35 0.44 0.45 0.45 0.44 0.34 0.43 0.45 0.45 0.45
0.37 0.35 0.36 0.35 0.35 1 0.46 0.45 0.44 0.44 0.36 0.35 0.35 0.35 0.35
0.35 0.42 0.44 0.44 0.44 0.46 1 0.66 0.67 0.66 0.34 0.42 0.43 0.43 0.43
0.34 0.43 0.45 0.45 0.45 0.45 0.66 1 0.76 0.76 0.33 0.42 0.44 0.44 0.44
0.34 0.43 0.45 0.45 0.45 0.44 0.67 0.76 1 0.79 0.32 0.43 0.44 0.44 0.44
0.33 0.43 0.45 0.44 0.44 0.44 0.66 0.76 0.79 1 0.32 0.42 0.44 0.45 0.44
0.36 0.34 0.34 0.34 0.34 0.36 0.34 0.33 0.32 0.32 1 0.45 0.43 0.42 0.43
0.34 0.42 0.43 0.44 0.43 0.35 0.42 0.42 0.43 0.42 0.45 1 0.65 0.65 0.64
0.33 0.43 0.45 0.45 0.45 0.35 0.43 0.44 0.44 0.44 0.43 0.65 1 0.75 0.75
0.33 0.43 0.45 0.45 0.45 0.35 0.43 0.44 0.44 0.45 0.42 0.65 0.75 1 0.78
0.33 0.43 0.45 0.46 0.45 0.35 0.43 0.44 0.44 0.44 0.43 0.64 0.75 0.78 1
5M 10M 20M 50M 100M 5M 10M 20M 50M 100M 5M 10M 20M 50M 100M
Sequencing depth
1
2
3
Replicate

Supplementary Figure 2 - Peak length distributions

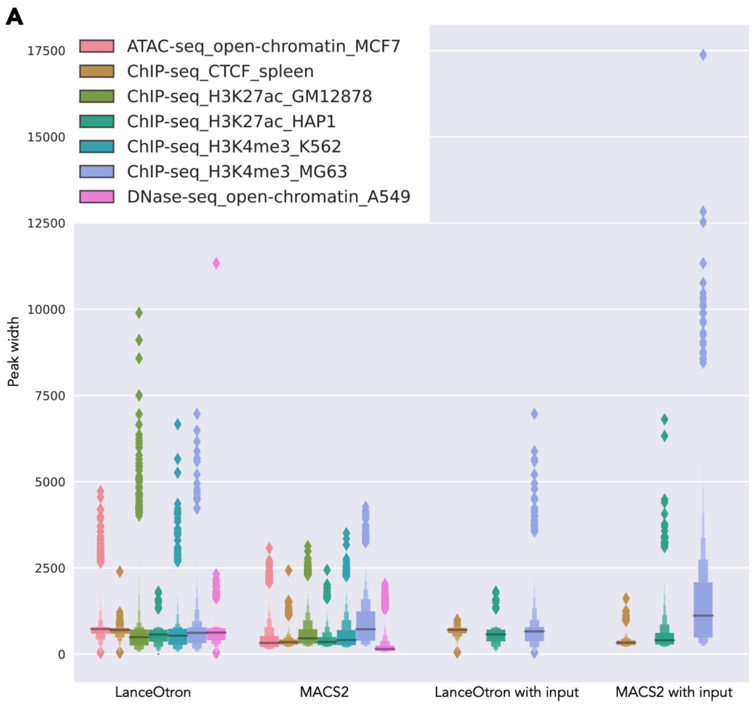

B

Average peak width (bp)	
LanceOtron	588.52
MACS2	393.42
LanceOtron with input	624.75
MACS2 with input	800.76

C

Total bases covered (Mb)	
LanceOtron	181.38
MACS2	192.53
LanceOtron with input	40.98
MACS2 with input	52.54

(A) Fragment length distribution data per benchmarking experiment (ATAC-seq in MCF-7 cells; CTCF ChIP-seq in spleen cells; H3K27ac ChIP-seq in GM12878 cells; H3K27ac ChIP-seq in HAP1 cells; H3K4me3 ChIP-seq in K562 cells; H3K4me3 ChIP-seq in MG63 cells; DNase-seq in A549 cells), grouped by peak caller. **(B)** Average peak width in base pairs (bp) over the benchmarking datasets, and **(C)** the total bases covered in megabases (Mb) .

Supplementary Figure 3 - LanceOtron performance compared to other MACS2 parameters

Performance benchmarking measuring precision, recall/sensitivity, selectivity, and overall F1 score for LanceOtron, LanceOtron-MACS2 hybrid, MACS2, MACS2-strict, and MACS2-broad - with and without input as available.

For LanceOtron-MACS2 and LanceOtron-MACS2 with input, the final peak call was generated by LanceOtron via the Score Peaks module, using a peak score of 0.5 as the threshold (consistent with all other benchmarking done). The candidate peak call was generated using MACS2 or MACS2 with input on default settings.

The MACS2-strict peak call was generated using a q-value of 0.01, and MACS2-broad used the broad option.

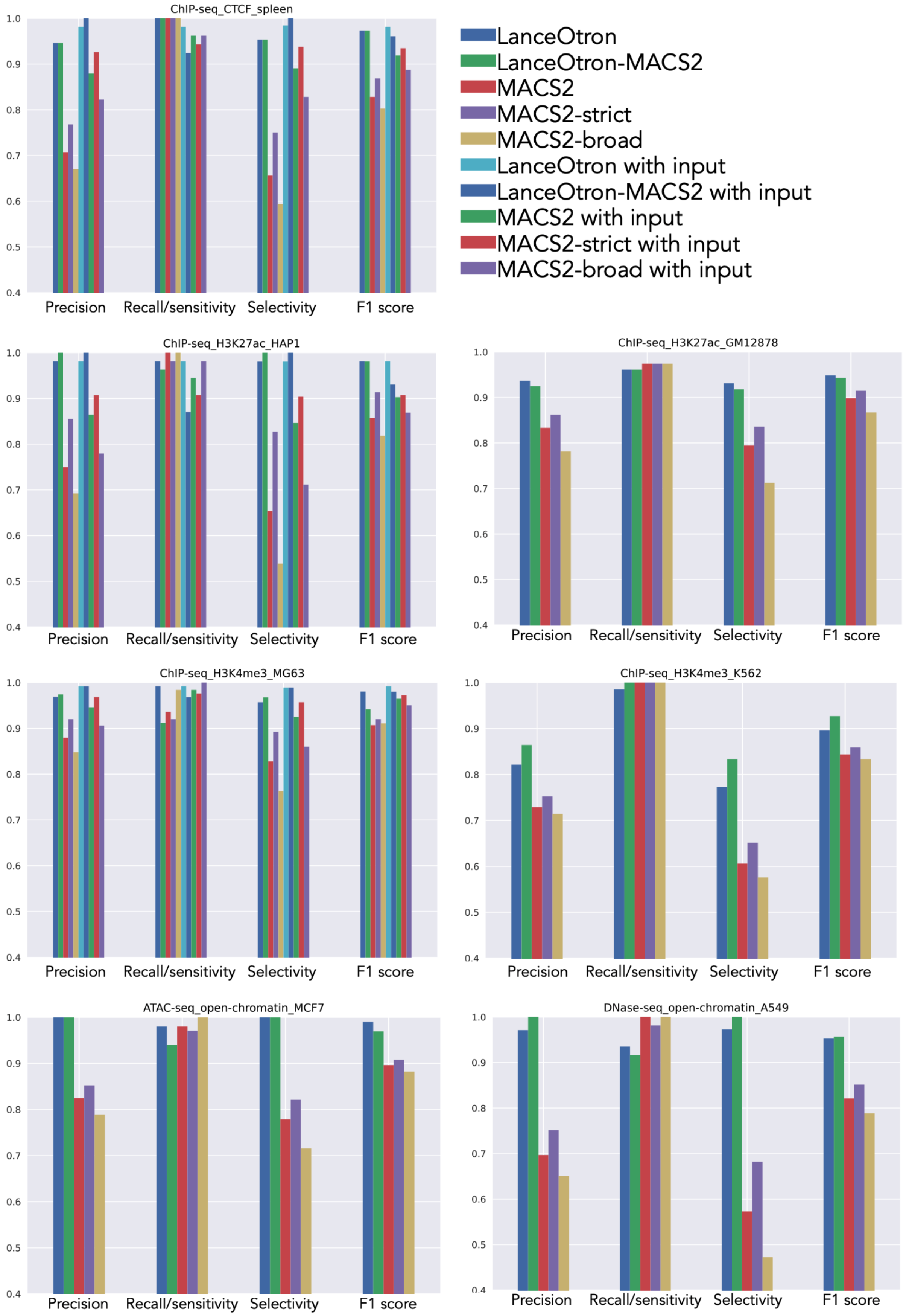
ChIP-seq_CTCF_spleen
LanceOtron
LanceOtron-MACS2
MACS2
MACS2-strict
MACS2-broad
LanceOtron with input
LanceOtron-MACS2 with input
MACS2 with input
MACS2-strict with input
MACS2-broad with input
ChIP-seq_H3K27ac_HAP1
ChIP-seq_H3K27ac_GM12878
ChIP-seq_H3K4me3_MG63
ChIP-seq_H3K4me3_K562
ATAC-seq_open-chromatin_MCF7
DNase-seq_open-chromatin_A549
Precision
Recall/sensitivity
Selectivity
F1 score
1.0
0.9
0.8
0.7
0.6
0.5
0.4

Supplementary Figure 4 - Gene associations for peaks missed by MACS2 but found with LanceOtron for SRF ChIP-seq in GM12878 cells

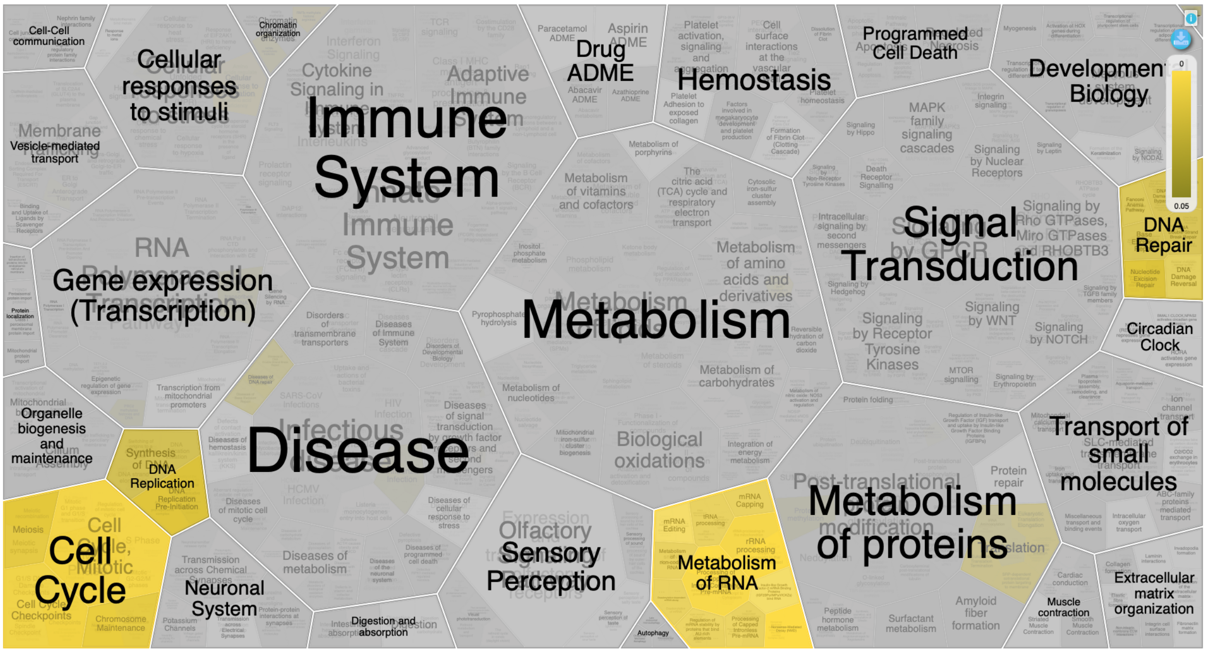

Image generated from Reactome (Fabregat *et al.*, 2017) showing gene enrichment, with brighter yellow representing more significance. Regions selected for analysis were chosen by peaks identified by LanceOtron only, and which were within 1 kb of a transcription start site.

Supplementary Figure 5 - Peak Score distribution across candidate peaks

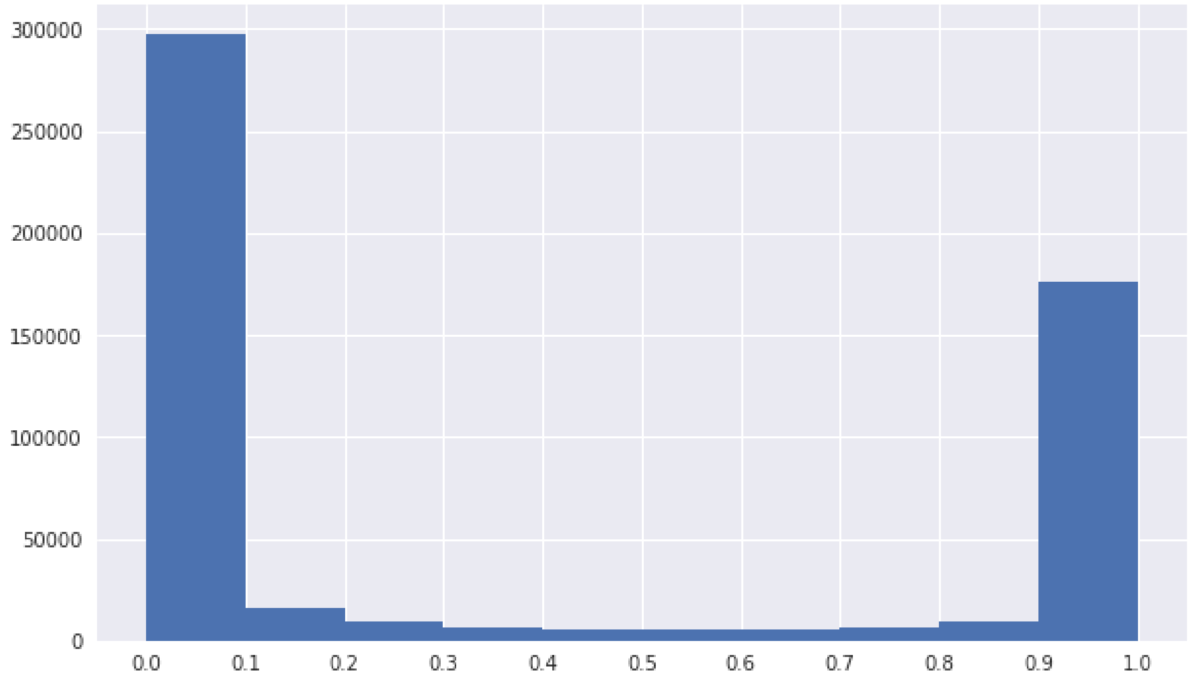

Histogram showing the total number of candidate peaks called by LanceOtron and their associated Peak Score over all in-house datasets. LanceOtron's deep learning model tends to score enriched regions being either peak or noise at high probabilities.

Supplementary Table 1 - ENCODE datasets used for training data and testing data

Training Data					
**Experiment**			**ENCODE ID numbers**		
**Assay**	**Target**	**Tissue**	**Experiment**	**BAM file**	**Control BAM**
ATAC-seq	Open chromatin	Breast epithelium	ENCSR955JSO	ENCFF656OYT	
ATAC-seq	Open chromatin	Tibial artery	ENCSR630REB	ENCFF168OTV	
ATAC-seq	Open chromatin	Foreskin keratinocyte	ENCSR290YMN	ENCFF799HAR	
ATAC-seq	Open chromatin	Adrenal gland	ENCSR113MBR	ENCFF436NOT	
ATAC-seq	Open chromatin	Foreskin keratinocyte	ENCSR158XTU	ENCFF784DSJ	
ATAC-seq	Open chromatin	Foreskin keratinocyte	ENCSR677MJF	ENCFF764CQI	
ATAC-seq	Open chromatin	Transverse colon	ENCSR668VCT	ENCFF377DAO	
ATAC-seq	Open chromatin	Sigmoid colon	ENCSR548QCP	ENCFF482HAC	
ATAC-seq	Open chromatin	Tibial nerve	ENCSR831KAH	ENCFF277DNH	
ATAC-seq	Open chromatin	Thyroid gland	ENCFF710ELD	ENCSR474XFV	
ChIP-seq	H3K27ac	RWPE1	ENCSR203KEU	ENCFF708CBX	ENCFF939LTT
ChIP-seq	H3K27ac	SKNSH	ENCSR564IGJ	ENCFF380OTV	ENCFF959FMO
ChIP-seq	H3K27ac	Bipolar neuron	ENCSR905TYC	ENCFF751YAL	ENCFF687LIL
ChIP-seq	H3K27ac	GM23338	ENCSR729ENO	ENCFF403VXK	ENCFF754UFV
ChIP-seq	H3K27ac	C42B	ENCSR279KIX	ENCFF913EZV	ENCFF980IJT
ChIP-seq	H3K27ac	22Rv1	ENCSR391NPE	ENCFF025ZEN	ENCFF769UET
ChIP-seq	H3K27ac	Foreskin keratinocyte	ENCSR709ABP	ENCFF085FAH	ENCFF178GZR
ChIP-seq	H3K27ac	Foreskin keratinocyte	ENCSR709ABP	ENCFF776HMQ	ENCFF178GZR
ChIP-seq	H3K27ac	Epithelial cell of prostate	ENCSR910PDW	ENCFF382XYO	ENCFF213AZI
ChIP-seq	H3K27ac	RWPE2	ENCSR987PNT	ENCFF245ORL	ENCFF169DGZ
ChIP-seq	H3K4me3	SKNSH	ENCSR975GZA	ENCFF027SGQ	ENCFF959FMO

ChIP-seq	H3K4me3	SKNSH	ENCSR975GZA	ENCFF245RXP	ENCFF959FMO
ChIP-seq	H3K4me3	NCIH929	ENCSR082NQB	ENCFF417RNS	ENCFF446RUP
ChIP-seq	H3K4me3	NCIH929	ENCSR082NQB	ENCFF067LLV	ENCFF446RUP
ChIP-seq	H3K4me3	Bipolar neuron	ENCSR849YFO	ENCFF096QTT	ENCFF687LIL
ChIP-seq	H3K4me3	Bipolar neuron	ENCSR849YFO	ENCFF950QWN	ENCFF687LIL
ChIP-seq	H3K4me3	Muscle of leg	ENCSR128QKM	ENCFF552OGD	ENCFF622XBJ
ChIP-seq	H3K4me3	Heart right ventricle	ENCSR107RDP	ENCFF897OOT	ENCFF246SXV
ChIP-seq	H3K4me3	Gastrocnemius medialis	ENCSR098OLN	ENCFF310NMI	ENCFF587DDD
ChIP-seq	H3K4me3	OCILY3	ENCSR548PZS	ENCFF816RLY	ENCFF691EEI
ChIP-seq	NR2C1	GM12878	ENCSR784VIQ	ENCFF785FLS	ENCFF322NTO
ChIP-seq	EP300	Ovary	ENCSR696LQU	ENCFF405UYE	ENCFF271JKY
ChIP-seq	NFXL1	GM12878	ENCSR746XEG	ENCFF673BXM	ENCFF322NTO
ChIP-seq	MXI1	Neural cell	ENCSR934NHU	ENCFF260PNL	ENCFF056HWK
ChIP-seq	ZNF318	K562	ENCSR334HSW	ENCFF373YTD	ENCFF790TAN
ChIP-seq	CREB1	HepG2	ENCSR112ALD	ENCFF011HOS	ENCFF950AXC
ChIP-seq	CTCF	RWPE1	ENCSR303GFI	ENCFF204KRO	ENCFF290UZX
ChIP-seq	RFX1	MCF7	ENCSR788XNX	ENCFF804LEF	ENCFF426RDP
ChIP-seq	CTCF	Ascending aorta	ENCSR960MDF	ENCFF353ZVY	ENCFF023NJF
ChIP-seq	E4F1	K562	ENCSR731LHZ	ENCFF978NVP	ENCFF910IKB
DNase-seq	Open chromatin	Left arm bone	ENCSR976XOY	ENCFF205JXZ	
DNase-seq	Open chromatin	A673	ENCSR346JWH	ENCFF348KWA	
DNase-seq	Open chromatin	T-helper 1 cell	ENCSR000EQC	ENCFF425YMJ	
DNase-seq	Open chromatin	Retina	ENCSR820ICX	ENCFF441YDL	
DNase-seq	Open chromatin	Uterus	ENCSR129BZE	ENCFF759POB	
DNase-seq	Open chromatin	NAMALWA	ENCSR301OGM	ENCFF554YJG	
DNase-seq	Open chromatin	SKMEL5	ENCSR000FEK	ENCFF844BZM	
DNase-seq	Open chromatin	ELF1	ENCSR678ILN	ENCFF433CFI	
DNase-seq	Open chromatin	Myocyte	ENCSR000EPD	ENCFF042QTI	
DNase-seq	Open chromatin	Pancreas	ENCSR828FVZ	ENCFF984FKS	

Supplementary Table 2 - ENCODE datasets used for testing data

**Testing Data**					
**Experiment**			**ENCODE ID numbers**		
**Assay**	**Target**	**Tissue**	**Experiment**	**BAM file**	**Control BAM**
ATAC-seq	Open chromatin	MCF-7	ENCSR422SUG	ENCFF346MIJ	
ChIP-seq	CTCF	Spleen	ENCSR692ILH	ENCFF903NKV	ENCFF376BTL
ChIP-seq	H3K27ac	HAP-1	ENCSR131DVD	ENCFF742SZS	ENCFF247DSQ
ChIP-seq	H3K4me3	MG63	ENCSR579SNM	ENCFF996ZSR	ENCFF381RWF
DNase-seq	Open chromatin	A549	ENCSR000ELW	ENCFF410CDT	

Supplementary Table 3 - numerical listing of performance benchmarks for all datasets

**CTCF ChIP-seq in spleen**										
	**LoTron**	**LoTron MACS2**	**MACS2**	**MACS2 strict**	**MACS2 broad**	**LoTron with input**	**LoTron MACS2 with input**	**MACS2 with input**	**MACS2 strict with input**	**MACS2 broad with input**
Precision	0.946	0.946	0.707	0.768	0.671	0.981	**1.000**	0.879	0.926	0.823
Recall / sensitivity	**1.000**	**1.000**	**1.000**	**1.000**	**1.000**	0.981	0.925	0.962	0.943	0.962
Selectivity	0.953	0.953	0.656	0.750	0.594	0.984	**1.000**	0.891	0.938	0.828
F1 score	0.972	0.972	0.828	0.869	0.803	**0.981**	0.961	0.919	0.935	0.887

H3K27ac ChIP-seq in HAP-1

	LoTron	LoTron MACS2	MACS2	MACS2 strict	MACS2 broad	LoTron with input	LoTron MACS2 with input	MACS2 with input	MACS2 strict with input	MACS2 broad with input
Precision	0.981	**1.000**	0.75	0.854	0.692	0.981	**1.000**	0.864	0.907	0.779
Recall / sensitivity	0.981	0.963	**1.000**	0.981	**1.000**	0.981	0.870	0.944	0.907	0.981
Selectivity	0.981	**1.000**	0.654	0.827	0.538	0.981	**1.000**	0.846	0.904	0.712
F1 score	**0.981**	**0.981**	0.857	0.914	0.818	**0.981**	0.931	0.903	0.907	0.869

H3K27ac ChIP-seq in GM12878

	LoTron	LoTron MACS2	MACS2	MACS2 strict	MACS2 broad					
Precision	**0.937**	0.925	0.833	0.862	0.781					
Recall / sensitivity	0.961	0.961	**0.974**	**0.974**	**0.974**					
Selectivity	**0.932**	0.918	0.795	0.836	0.712					
F1 score	**0.949**	0.943	0.898	0.915	0.867					

H3K4me3 ChIP-seq in MG63

	LoTron	LoTron MACS2	MACS2	MACS2 strict	MACS2 broad	LoTron with input	LoTron MACS2 with input	MACS2 with input	MACS2 strict with input	MACS2 broad with input
Precision	0.969	**0.974**	0.880	0.920	0.848	0.992	0.992	0.946	0.968	0.906
Recall / sensitivity	0.992	0.912	0.936	0.920	0.984	0.992	0.968	0.984	0.976	**1.000**
Selectivity	0.957	0.968	0.828	0.892	0.763	**0.989**	**0.989**	0.925	0.957	0.860
F1 score	0.980	0.942	0.907	0.920	0.911	**0.992**	0.980	0.965	0.972	0.951

H3K4me3 ChIP-seq in K562

	LoTron	LoTron MACS2	MACS2	MACS2 strict	MACS2 broad					
Precision	0.821	**0.864**	0.729	0.753	0.714					
Recall / sensitivity	0.986	**1.000**	**1.000**	**1.000**	**1.000**					
Selectivity	0.773	**0.833**	0.606	0.652	0.576					
F1 score	0.896	**0.927**	0.843	0.859	0.833					

ATAC-seq in MCF-7

	LoTron	LoTron MACS2	MACS2	MACS2 strict	MACS2 broad					
Precision	**1.000**	**1.000**	0.825	0.852	0.789					
Recall / sensitivity	0.980	0.941	0.980	0.970	**1.000**					
Selectivity	**1.000**	**1.000**	0.779	0.821	0.716					
F1 score	**0.990**	0.969	0.896	0.907	0.882					
**DNase-seq in A549**										
	**LoTron**	**LoTron MACS2**	**MACS2**	**MACS2 strict**	**MACS2 broad**					
Precision	0.971	**1.000**	0.697	0.752	0.651					
Recall / sensitivity	0.935	0.917	**1.000**	0.981	**1.000**					
Selectivity	0.973	**1.000**	0.573	0.682	0.473					
F1 score	0.953	**0.957**	0.821	0.851	0.788					

Supplementary Table 4 - motif enrichment analysis of transcription factors

**CTCF in spleen**				
	**LanceOtron**	**MACS2**	**LanceOtron with input**	**MACS2 with input**
Peaks called	19291	28953	17418	18390
Peaks called with motifs	2639	7538	1675	1878
% Peaks with motifs	86.3	74.0	**90.4**	89.79
**GATA1 in primary erythroid**				
	**LanceOtron**	**MACS2**	**LanceOtron with input**	**MACS2 with input**
Peaks called	3227	4587	2954	4467
Peaks called with motifs	1446	1744	1420	1782
% Peaks with motifs	44.8	38.0	**48.1**	39.9

ATF2 in spleen				
	**LanceOtron**	**MACS2**	**LanceOtron with input**	**MACS2 with input**
Peaks called	53807	89390	53750	77469
Peaks called with motifs	28734	39953	28734	37403
% Peaks with motifs	53.4	44.7	**53.5**	48.3

REST in spleen				
	**LanceOtron**	**MACS2**	**LanceOtron with input**	**MACS2 with input**
Peaks called	51508	74803	49669	55288
Peaks called with motifs	30516	36312	30069	30615
% Peaks with motifs	59.2	48.5	**60.5**	55.4

SRF in spleen				
	**LanceOtron**	**MACS2**	**LanceOtron with input**	**MACS2 with input**
Peaks called	9766	20469	9034	7198
Peaks called with motifs	3785	5493	3730	3634
% Peaks with motifs	38.8	26.8	41.3	**50.5**

Supplementary Table 5 - Transcription start site enrichment analysis of SRF

SRF in spleen				
	**LanceOtron**	**MACS2**	**LanceOtron with input**	**MACS2 with input**
Peaks called	9766	20469	9034	7198
Peaks called intersecting TSSs	3655	5779	3202	1498
% Peaks intersecting TSSs	**37.4**	28.2	35.4	20.4

Functionality of LanceOtron's user interface

LanceOtron features a rich graphical user interface, accessible using any web browser, and allows peak calls to be made without the use of the command line. Using the web tool to perform a peak call is demonstrated in **supplementary video 1**: https://youtu.be/k8GrIp55vDg. Furthermore, exploring and filtering data is also easily carried out with the graphical interface, demonstrated in **supplementary video 2**: https://youtu.be/M5ox8XI-U4Q.

References

Cheng,H.-T. *et al.* (2016) Wide & Deep Learning for Recommender Systems. *Proceedings of the 1st Workshop on Deep Learning for Recommender Systems - DLRS 2016*.

Fabregat,A. *et al.* (2017) Reactome pathway analysis: a high-performance in-memory approach. *BMC Bioinformatics*, **18**, 142.

Li,H. *et al.* (2009) The Sequence Alignment/Map format and SAMtools. *Bioinformatics*, **25**, 2078–2079.

Quinlan,A.R. and Hall,I.M. (2010) BEDTools: a flexible suite of utilities for comparing genomic features. *Bioinformatics*, **26**, 841–842.

Ramírez,F. *et al.* (2014) deepTools: a flexible platform for exploring deep-sequencing data. *Nucleic Acids Res.*, **42**, W187–91.

Sergeant,M.J. *et al.* (2021) Multi Locus View: an extensible web-based tool for the analysis of genomic data. *Commun Biol*, **4**, 623.
